# Integrating sentiment and social structure to determine preference alignments: the Irish Marriage Referendum

**DOI:** 10.1098/rsos.170154

**Published:** 2017-07-12

**Authors:** David J. P. O’Sullivan, Guillermo Garduño-Hernández, James P. Gleeson, Mariano Beguerisse-Díaz

**Affiliations:** 1 MACSI, Department of Mathematics and Statistics, University of Limerick, Limerick, Ireland; 2 Sinnia, Mexico City, Mexico; 3 Mathematical Institute, University of Oxford, Oxford OX2 6GG, UK

**Keywords:** online social media, networks, text analysis, sentiment, public opinion, homophily

## Abstract

We examine the relationship between social structure and sentiment through the analysis of a large collection of tweets about the Irish Marriage Referendum of 2015. We obtain the sentiment of every tweet with the hashtags #marref and #marriageref that was posted in the days leading to the referendum, and construct networks to aggregate sentiment and use it to study the interactions among users. Our analysis shows that the sentiment of outgoing mention tweets is correlated with the sentiment of incoming mentions, and there are significantly more connections between users with similar sentiment scores than among users with opposite scores in the mention and follower networks. We combine the community structure of the follower and mention networks with the activity level of the users and sentiment scores to find groups that support voting ‘yes’ or ‘no’ in the referendum. There were numerous conversations between users on opposing sides of the debate in the absence of follower connections, which suggests that there were efforts by some users to establish dialogue and debate across ideological divisions. Our analysis shows that social structure can be integrated successfully with sentiment to analyse and understand the disposition of social media users around controversial or polarizing issues. These results have potential applications in the integration of data and metadata to study opinion dynamics, public opinion modelling and polling.

## 1. Introduction

The Republic of Ireland held a referendum to legalize same-sex marriage on 22 May 2015. This referendum saw a high turnout (60.52% of voters), and the final result was a 62% majority in favour of the legalization of same-sex marriage. Such a high turnout represented a dramatic increase compared with previous referenda [1]. The enthusiasm of the electorate was reflected in the activity of online social media platforms, particularly Twitter, which saw a wealth of activity in the days preceding the referendum [2].

Twitter is an online micro-blogging platform where users can post short messages or *tweets* that can be up to 140 characters long; in Ireland, an estimated 25% of adults have a Twitter account, of which 36% use the service every day [3]. Users can subscribe to other users’ tweets (or *follow*); such following relationships are often asymmetric: if one user follows another, a reciprocated following relationship does not always exist [4]. In addition to following each other, there are other ways in which users can publicly interact such as *re-tweeting* (passing forward another user’s tweet), and mentioning each other in tweets. Twitter has been a popular venue for the dissemination of information, memes and opinions, and has facilitated public debate about a variety of subjects [4–11]. As a result, Twitter has received considerable attention from researchers who wish to gain insights into the relationships and mechanisms that govern these social interactions [12].

The use of sentiment analysis to infer the disposition of individuals or groups towards specific topics is a growing area of interest in computational social science [12–17]. For example, sentiment analysis on Twitter data has been used to study stock market fluctuations [18,19], film box-office performance [20] and reviews [21], tracking the spread of influenza [22] and (albeit controversially) predicting elections [10,23–26]. Although some of these studies have well-noted shortcomings [27,28], the idea of using the content of tweets to gain insight into social phenomena remains a promising and compelling one. Recent studies, using carefully constructed methodologies, have successfully leveraged sentiment to uncover insights into its effect on the spreading of cascades on Twitter [9], and how top broadcasters send messages with positive sentiment more often than negative [29].

The amount of Twitter activity during the Irish Marriage Referendum thus provides an excellent opportunity to understand how users interact around controversial or polarizing topics. A feature of the referendum which facilitates its analysis is that it posed a clear yes/no question compared to other, more complex consultations where voters must rank a range of options (e.g. general elections in Ireland). Furthermore, the perceived polarizing nature of the referendum can lead to easily distinguishable camps supporting voting *yes* or *no*, which is more tractable than, for example, attempting to assign members of the electorate to a political party in the presence of many similar political groups. In this work, we combine analyses of sentiment and social structure to explore Twitter conversations about the Irish marriage referendum. In particular, we address the following questions:

— How did Twitter users interact with each other in the context of the Irish Marriage Referendum?
— Can user interactions and the sentiment of their tweets help us find supporters of voting *yes* (in favour of the legalization of same-sex marriage) and *no* (against it)?

To answer these questions, we analyse an extensive dataset of tweets about the referendum, and the interactions among the users who posted the tweets (§2). We extract a sentiment score for each tweet (§3), and incorporate it into the structure of the mention and follower networks of users (§4). These networks enable the analysis of how the sentiment of users is correlated, and the proclivity of users with positive/negative sentiment to cluster together (§5). We use community detection to partition the users in the mention and follower networks into groups who communicate more or are generally more interested in each other’s content. We examine these communities from the vantage point of sentiment analysis to find a parsimonious three-group partition of the users (§6). These three groups are broadly composed of *yes* and *no* supporters with varying levels of activity, and starkly different patterns of interaction with each other (§7). Finally, in §8 we discuss our results and explore potential future research directions.

## 2. Data

**Figure 1.**
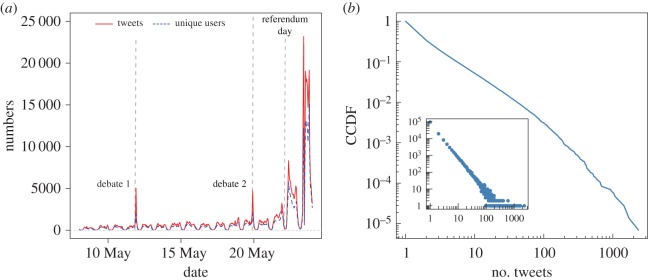
(*a*) Number of tweets containing the hashtags #marref and #marriageref (solid red line) and number of unique users (dashed blue line) in 15-min bins. The volume of tweets increases over time with the notable spikes for the two televised debates and the referendum day. (*b*) Complementary cumulative distribution function (CCDF) for number of tweets per user on a log–log scale (inset: the probability distribution function (PDF) of the same data).

The dataset we analyse in this work consists of *every* tweet containing the hashtags #marref and #marriageref from 8 May to 23 May 2015 (one day after the referendum). In total, we collected 499 642 tweets posted by 144 007 unique users (figure 1*a*). A total of 204 626 tweets were posted before the referendum day; 88 320 on the day and 206 696 after. The peaks observed in figure 1*a* coincide with the first and second televised debates (held on May 11 and May 19) and the referendum day (May 22), the tallying and announcement of the results, and subsequent global reaction. Figure 1*b* shows that the number of tweets per user has a heavy tailed distribution. The vast majority of users only posted a small number of tweets with the tracked hashtags, while a small number of users are responsible for a large volume of tweets. Of the total number of tweets, 135 370 (27%) were *original*, 24 397 (5%) were *replies* and 339 875 (68%) were *retweets*. Broadly speaking, original tweets are messages that are not in response to another previously posted tweet (i.e. the content is ‘new’), replies are tweets that are posted in response to an existing original tweet and retweets are tweets written by others that a user passes along to his/her followers. Users can *mention* each other in their tweets by inserting a user’s screen name (technically, replies and retweets can be seen as specific types of mention tweets). In our data, there are 388 161 mention tweets (78% of all tweets), of which 25 732 are original, 23 131 are replies and 339 298 are retweets. In addition to tweets, we also obtained the follower relationships of all users who used at least one of the hashtags (i.e. a list of everyone who is followed by the authors of the tweets in our data, regardless of whether they used the tracked hashtags). These correspond to 117 669 550 follower links. We also collected user information such as self-defined location, self-description and how long the user has been a member of Twitter.

All data were collected by Sinnia, a data analytics company, using Twitter Gnip Power-Track API^1^ which returns a complete dataset, not just a sample [30]. Using the Twitter stream API has the limitation that as the popularity of a search term (e.g. a hashtag) increases, the representativeness of the sample decreases [31]. By extracting *all* tweets with the two hashtags and all user-following relationships, we are able to circumvent such sampling issues. There could be, however, other issues with the data. For example, it is possible that our data gathering could miss important tweets or individuals if they never tweeted using one of the tracked hashtags. However, due to the ubiquity of the hashtags #marref and #marriageref in the weeks leading up to the referendum, we are confident that our data are an adequate representation of the Twitter discourse about the topic.

## 3. Sentiment of tweets

To quantify the positive or negative emotions of a tweet, we compute its sentiment score. We do not consider sentiment with the categorical *positive* or *negative* labels; instead we consider sentiment to be a number whose magnitude denotes how positive or negative the language expressed is [32]. For this task, we use the open source sentiment algorithm *SentiStrength*, a lexicon-based sentiment algorithm that searches for words that have an associated positive or negative score [33]. SentiStrength provides a score of both the positive *and* the negative emotional charge of a string of text (in this case, of each tweet in our data). Positive scores range from 1 to 5, and negative scores from −1 to −5. A score of 1 (or −1) indicates that the tweet has no positive (or negative) sentiment, while a score of 5 (or −5) means that the tweet has the maximum positive (negative) score possible. See appendix A for more details on how sentiment scores are obtained with SentiStrength.

**Figure 2.**
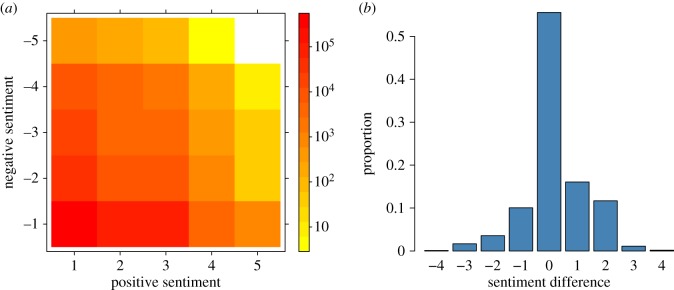
(*a*) Density plot of the two-dimensional sentiment scores of all tweets. (*b*) Histogram of the difference between the positive and negative score of each tweet.

Figure 2*a* shows the two-dimensional distribution of sentiment scores of all the tweets in our dataset. To simplify calculations, we add the positive and negative scores of each tweet to obtain a one-dimensional score between −4 and 4. A negative score indicates that the tweet contains stronger negative language than positive, and vice versa for positive scores. Figure 2*b* shows the distribution of the unidimensional sentiment scores of all tweets in the dataset. About half of all tweets (55%) have a score of zero; of these, the vast majority (95%) have a score of 1 and −1 for positive and negative language, respectively (i.e. no detected sentiment), and the rest have balanced positive and negative sentiment scores. The distribution is roughly symmetric around zero with a slight positive skew; this observation is consistent with previous reports of sentiment bias in language [16] and tweets [29]. As noted in appendix A, the SentiStrength scores of a single tweet can be unreliable, so a single tweet does not provide definitive information about the user’s sentiment. To obtain a more robust indication of users’ sentiment, we aggregate the scores of all the tweets produced by one user to obtain a single score. Although aggregate scores can help overcome some issues, computing a single score per author neglects the fact that Twitter users often interact with multiple people, and that the sentiment of these interactions may vary substantially depending on the counterpart and the nature of the exchange. Therefore, using exclusively a single score per user can lead to information loss, and provide a misleading indication about the user’s sentiment. To avoid these problems, it is necessary to incorporate the users’ interactions into the analysis.

## 4. Sentiment aggregation and social structure

Although SentiStrength has been reported to perform well on Twitter datasets [33], the nuances and complexities of human language (for example, sarcasm, idioms, negation, double negatives and a cavalier attitude towards grammar) make the process of automatically extracting sentiment a challenging task. In addition, Twitter users do not exist in isolation, they interact with each other through mentions, replies and friend/follower relationships. For this reason, it is necessary to incorporate social structure to obtain a more robust description of the user’s disposition with regard to the marriage referendum. We focus our analysis on two types of Twitter networks:

(i) Reciprocal mention network: Connections exist between users who have mentioned each other in tweets containing the tracked hashtags.
(ii) Reciprocal follower network: Connections exist between users who *follow* each other on Twitter.

The information contained in these networks reflects complementary aspects of the interactions between users: the reciprocated mention network includes interactions that arise *specifically* from conversations about the Irish marriage referendum, and are constrained to the observation period (8–23 May 2015). We are interested in studying reciprocal mentions because they are a sign of genuine interactions between users [29,34]. By contrast, the follower network is not constrained to discussions about the marriage referendum, nor to the observation period; this network provides a broader view of how users are interested in each other. Table 1 provides a summary of statistics for both networks.

**Table 1.** Summary statistics for the mention and follower networks.

	mention		follower	
	full	reciprocal	full	reciprocal
nodes	40 812	2047	36 674	2047
links	227 203	69 022	3 309 687	173 137
reciprocal links	23 713	22 218	1 398 236	85 986
avg. out-degree	9	34	90	85
transitivity	0.02	0.13	0.09	0.28

By restricting the analysis to users who have made reciprocal mentions, we do exclude a large number of users (table 1). Although there are fewer users with reciprocal mentions, they have a higher average out-degree (number of mention tweets written) than in the full network (34 in the reciprocal group compared with nine in the general population), which enables a more robust analysis of their (noisy) sentiment scores. A possible drawback of focusing on reciprocal mentions is the introduction of a selection bias. By excluding less-active users, the population of those who have not often expressed their beliefs or engaged in the debate may be under-represented, in particular users who favoured the less popular *no* position.

### 4.1. Construction of the networks

We construct the directed mention network by searching each user’s tweets for mentions of other users (indicated by a prefixed ‘@’). A mention often indicates that the author wishes to draw the attention of another user to the content of the tweet; this could be original content directed at a user, a retweet or a reply. The announcement of the referendum results received widespread international attention, which translated into a large number of tweets from users outside of Ireland (figure 1*a*). We are specifically interested in detecting *yes* and *no* supporters, which is why we further refine our networks to only include tweets generated before the day of the referendum. Each mention creates a directed connection from the author of the tweet to the user it mentions. We incorporate sentiment into this network by setting the weight of the connection to be the sentiment score of the tweet. When there are multiple directed mentions, we average their sentiment scores. The resulting network is directed, weighted and signed (negative weights indicate when the mentions have a predominantly negative sentiment); it contains 40 812 unique users and 227 203 directed connections. Note that some users who appear in this network may not have used one of the tracked hashtags; they only need to have been mentioned in a tweet containing one of them. The average combined in- and out-degree is 11, with a transitivity coefficient of 0.02 (based on treating links as undirected). The *reciprocal mentions network* is the subnetwork in which connected individuals have mentioned *each other* in their tweets at least once. This network has 2830 users with non-zero in- and out-degree, and 23 713 edges (approx. 10% of the mentions in the full network).

In the follower network, a directed connection denotes that the source of the connection ‘follows’ the target on Twitter, so the in-degree is the number of followers and the out-degree is the number of people followed by the user. To construct this network, we obtain the following relationships between users who authored the tweets in our dataset. This network has 36 674 users with 3 309 687 unweighted connections, of which 1 398 236 (42%) are reciprocal. Note that the follower network is unweighted. The average combined in- and out-degree is 180 and the transitivity coefficient is 0.09. The full follower network has a different size from that of the full mention network because the latter network’s starting point was the users who have authored at least one of the tweets in our database. The reciprocal mention network has 2830 of which 2056 are in the largest connected component. Of these users, 2047 users are in the largest connected component of the follower network. The final mention and follower networks contain the users in this 2047 node set with 69 022 and 173 137 connections, respectively.

**Figure 3.**
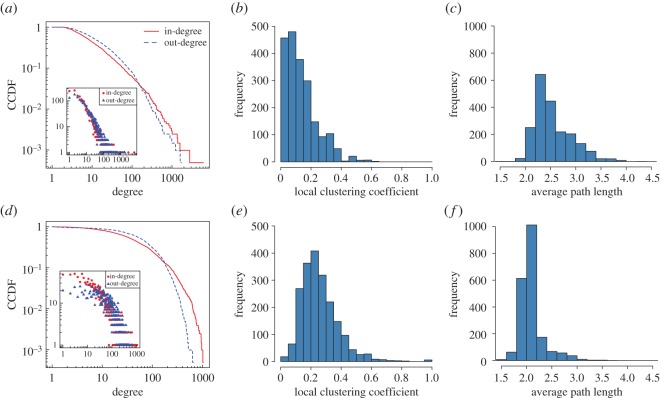
Descriptive network statistics of the mention (*a*–*c*) and follower (*d*–*f*) networks: the CCDF for the in- and out-degree distribution (*a*,*d*), distribution of local clustering coefficients (*b*,*e*), and average path length distribution (*c*,*f*) for the reciprocal mention and follower network, respectively. (Insets in (*a*,*d*): the PDFs for the same data).

Table 1 contains the global summary statistics of the networks. Figure 3 shows that the in- and out-degree distribution in the mention network appear to be similar; most users only sent and received a small number of mention tweets (figure 3*a*). By contrast, the in- and out-degree distribution on the follower network is much less heavy-tailed; many users here have a large number of friends and followers. The local clustering coefficient (based on treating links as undirected) in the mention network is between zero and 0.33 (5% and 95% percentiles), and 0.10 and 0.48 in the follower network (figure 3*b*,*e*). In the mention network, the distribution is peaked closer to zero than in the follower network (the means are 0.14 and 0.26, respectively); in other words, the interactions in the mention network are less transitive than in the follower network. Similarly, in both the mention and follower networks, the average (undirected) path length between users is between 2.11 and 3.23 (5% and 95% percentiles), and 1.83 and 2.62, respectively (figure 3*c*,*f*). This distribution in the mention network is peaked around its mean of 2.53 with a slight right skew, and the path length distribution in the follower network around its mean of 2.09.

To incorporate the sentiment of tweets with the social structure of the networks, we compute four user attributes: the average in- and out-sentiment (*S*_I_ and *S*_O_) of each user in the mention network, as well as the average in- and out-sentiment of each user’s neighbours (*S*^n^_I_ and *S*^n^_O_). These quantities allow us to aggregate sentiment scores while preserving the heterogeneity of the user’s interactions (e.g. supportive or adversarial discussions).

**Figure 4.**
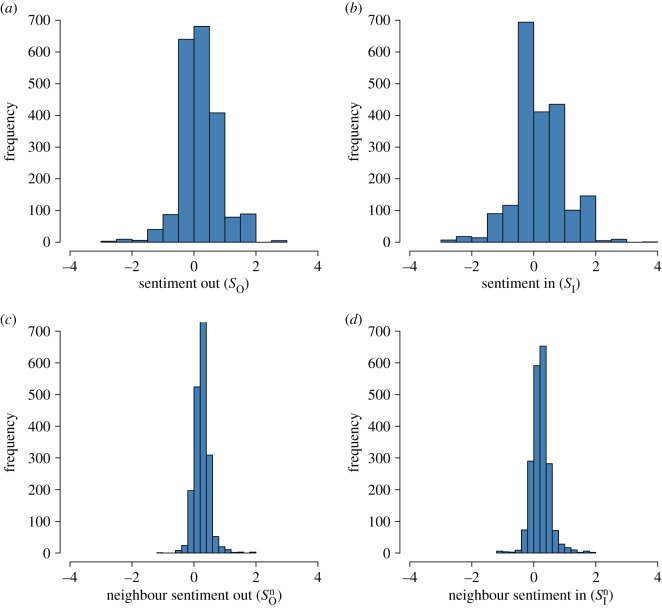
Distribution of the average of users’ (*a*) in-sentiment, (*b*) out-sentiment, (*c*) neighbours’ in-sentiment and (*d*) neighbours’ out-sentiment.

Figure 4*a*,*b* shows that the distributions of average user in- and out-sentiment are similar. The average out-neighbour sentiment is marginally higher than the average in-neighbour sentiment (0.26 versus 0.22, see figure 4*c*,*d*). These distributions are approximately symmetric around their mean with a slight skew to the right.

## 5. User sentiment alignment

As discussed in §3 (and appendix A), the sentiment score of a single tweet is not entirely reliable; however, the scores of a large number of tweets can provide a more robust indication of the sentiment of the corpus. We test this notion against the null hypothesis that the sentiment is generated by an inherently random process: for example, if the content of the tweets is completely unrelated to sentiment, or if the sentiment extraction process gives noisy scores that do not contain any information about the actual sentiment of the tweets.

More precisely, we seek to determine (1) whether user in- and out-sentiment scores are correlated, and (2) if users whose tweets have similar sentiment tend to be clustered in the network. If the sentiments of the mention tweets that a user sends and receives are correlated, and users tend to cluster together with others with similar sentiment, we could then consider sentiment alignment as a proxy for homophily among users. We can reasonably expect this because users with a similar disposition towards the referendum may communicate using similar language. For instance, *yes* campaigners may use phrases that are more positively charged (e.g. ‘vote yes’) more often in their tweets, which results in a higher positive user sentiment (and vice versa for *no* campaigners).

To answer (1), we examine whether there is a correlation between a user’s in- and out-sentiment. The Pearson correlation between *S*_I_ and *S*_O_ is 0.60, which indicates a moderate linear relationship between these two nodal attributes [35]. To confirm that this correlation is not due to chance alone, we use a procedure based on redistributing the sentiment of a user’s tweets. The randomization procedure is as follows:

— Sample a sentiment score for each connection from the observed distribution of link scores with replacement. This keeps the network topology intact.
— Calculate the average randomized in- and out-sentiment of each user (*S*^r^_I_ and *S*^r^_O_).
— Calculate the correlation coefficient between *S*^r^_I_ and *S*^r^_O_ in the resampled network.

**Figure 5.**
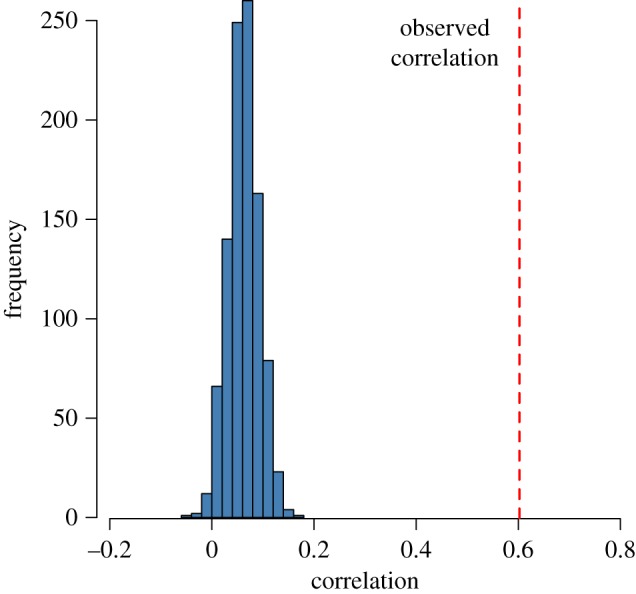
Distribution of the correlation between *S*^r^_I_ and *S*^r^_O_ after 1000 randomizations (blue bars), and the observed correlation between *S*_I_ and *S*_O_ in the data (red dashed line).

Figure 5 shows the comparison of the resulting distribution of the correlation between (*S*^r^_I_ and *S*^r^_O_) after 1000 iterations of the procedure with the observed correlation of *S*_I_ and *S*_O_ in our data. This result indicates that there is a non-trivial correlation between the sentiment of what a user tweets and receives.

**Figure 6.**
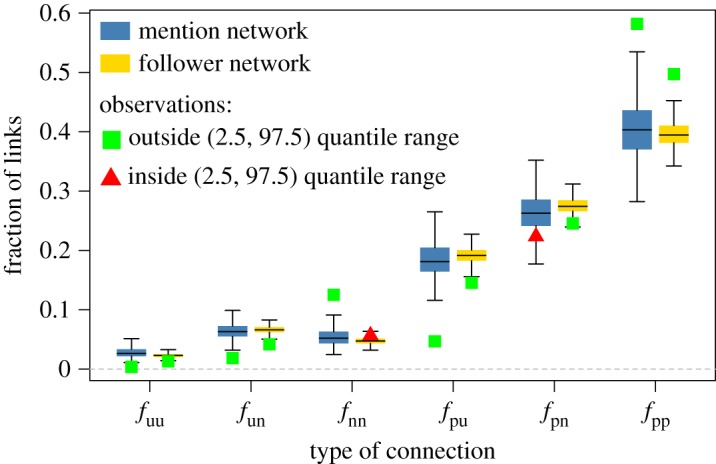
Result of the randomization tests in the mention (blue box plots) network and the follower network (yellow box plots). The green squares and red triangles mark the observed fraction of links in the data. Green squares indicate that the observed fraction of connections falls outside the lower 2.5% and upper 97.5% quantiles of the randomized distribution (i.e. it is unlikely to arise by chance alone); red triangles indicate that the observed fraction falls inside the lower 2.5% and upper 97.5% quantiles of the randomized distribution.

To answer (2), we investigate whether users with similar sentiment are clustered together in the mention and follower networks. The observed correlation between *S*_I_ and *S*_O_ suggests that users may be more likely to be connected to other users with similar sentiment scores. We create three coarse class labels for users according to their sentiment—aggregate scores above zero are ‘positive’, scores less than zero are ‘negative’ and scores equal to zero are ‘unknown’—and we find the fraction of links connecting users of these broad sentiment labels. We denote the fraction of links between positive and positive users as *f*_pp_, the fraction of links between positive and negative users as *f*_pn_, between positive and unknown users as *f*_pu_, and so on. In total, there are six types of links: *f*_pp_, *f*_pn_, *f*_pu_, *f*_nn_, *f*_un_ and *f*_*uu*_. We randomize the class labels of each user by sampling from the observed distributions with replacement, and recalculate the fraction of connections; we repeat this process 1000 times. As before, we compare the randomized distributions of the fractions with the observed fraction in our data; figure 6 shows the results obtained from this procedure.

The randomization test in the mention network (blue box plots in figure 6) shows that it is highly unlikely that the observed values of *f*_pp_, *f*_pu_, *f*_nn_, *f*_un_ and *f*_uu_ in the mention network arise from chance. There are fewer connections involving unknown users ( *f*_un_ and *f*_uu_ and *f*_pu_) than we would expect by chance. By contrast, the connections between positive users ( *f*_pp_) and negative users ( *f*_nn_) are higher than expected. The fraction of connections between positive and negative users ( *f*_pn_) is less than what we would expect (below the 25% quantile), although this result is less robust than the rest. This analysis shows that users tend to mention others with similar sentiment in their tweets more frequently than we would expect by chance. The same analysis in the follower network (yellow box plots in figure 6) paints a broadly consistent picture. We find more links between positive users, fewer links between positive and negative, and fewer links involving unknown users than we would expect by random chance.

This analysis provides evidence of a relationship between users’ *S*_I_ and *S*_O_, and their preference to engage with users of a similar sentiment, and supports the intuition that in this case sentiment can be a proxy for homophily. Labelling users as positive, negative or unknown according to the sign of their *S*_O_ may seem too coarse; to test this, we performed a similar analysis in which we split the users according to their quantile, or by above/below the mean or median (see appendix C). These tests produced very similar results to those presented in this section.

## 6. Communities and sentiment

**Figure 7.**
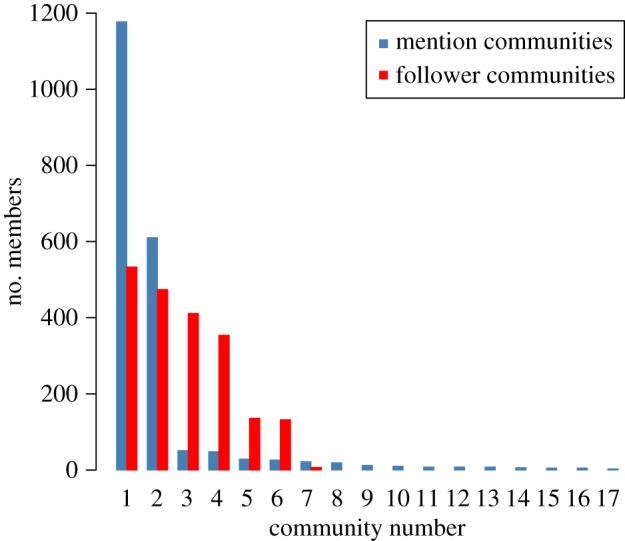
Number of users in each community in the mention (blue) and follower (red) networks.

We are interested in finding groups of users that are not only tightly connected in both networks, but also whose tweets have similar sentiment. For this task, we extract the communities in each network, and enrich the partition with the analysis of connection patterns from the previous section. We use Markov Stability [36,37] to obtain a robust partition of the mentions network into 17 communities, and a partition of the follower network into seven communities (figure 7).

The communities in the mention network arise specifically from conversations between users; the links consist of mention tweets containing the tracked hashtags posted during the observation period. The communities in the follower network arise from users’ declared interests in receiving tweets from others, which may not necessarily be restricted to the marriage referendum. Note that, although the mention network has 17 communities, two of them contain the overwhelming majority of the users. By contrast, users are more evenly distributed in the seven communities in the follower network.

**Figure 8.**
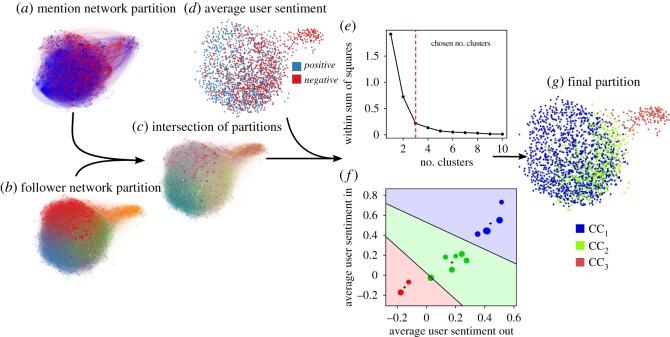
Schematic of the analysis of communities and sentiment. Communities in the mention network (*a*) and follower (*b*) networks. The intersection of the communities in both networks is shown in (*c*). Mention network with nodes coloured according to sentiment (*d*). The *k*-means clustering of the sub-communities according to their sentiment reveals three broad clusters (*e*). The relationship between in- and out-sentiment of each sub-community and cluster membership is shown in (*f*). The size of each marker is proportional to the size of each sub-community; crosses indicate the centroid of each cluster. (*g*) Final partition of users into three ‘community clusters’ in the mention network.

Now, we seek a new grouping of users based on both partitions, and use the sentiment scores to construct a measure of similarity. To accomplish this task, we intersect the partitions of the two networks to obtain 62 sub-communities (figure 8*c*). Each of these new groups contains users that are in the same community in both networks; these users are not only more broadly interested in each other (because they follow each other), but also had conversations about the referendum. Then, we calculate the average in- and out-sentiment and neighbour sentiment ${{\bar{S}}_{I}}_{i}$, ${{\bar{S}}_{O}}_{i}$, ${{\bar{S}}_{I}^{n}}_{i}$ and ${{\bar{S}}_{O}^{n}}_{i}$ in each sub-community *i*∈{1,…,62}. As we noted in the previous section, we consider sentiment as a proxy for homophily between users; therefore we use aggregate sentiment scores as an indication of similarity between the 62 sub-communities. However, 49 of these sub-communities have 20 users or fewer (224 users in total). Because sentiment scores of individual tweets are a noisy signal and these communities are small, we are unable to provide a robust statistical description in these communities. To limit the effect of this noise, we remove these sub-communities and proceed to analyse the remaining 13 sub-communities. This procedure is illustrated in figure 8.

We use *k*-means clustering to group the sub-communities according to the Euclidean distance between the average in- and out-sentiment and neighbour sentiment scores of each sub-community. To choose the number of clusters, we locate the bend in the plot of the total within-sum-of-squares sentiment difference of the members of the groups (figure 8*e*). A marked flattening of the graph suggests that a finer clustering is not considerably better at segregating sub-communities into distinct groups than a more parsimonious clustering with fewer groups. The appropriate number of clusters is found at the ‘elbow’ of the graph [38], which in this case is three. Figure 8*f* shows the three regions in which we have classified the sub-communities. We call these clusters of sub-communities *community clusters*: CC_1_ with 1064 users, CC_2_ with 604 and CC_3_ with 155. Community cluster CC_1_ has the highest in- and out-sentiment, followed by CC_2_ and CC_3_.

**Figure 9.**
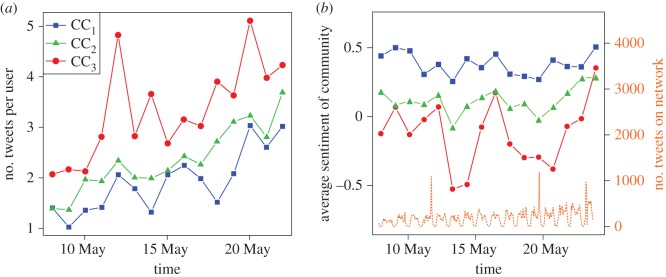
(*a*) Average number of tweets per user per day in each community cluster. (*b*) Out-sentiment of each community cluster over time (left *y*-axis) and number of tweets (right *y*-axis, orange dotted line).

Table 2 contains the summary statistics for each of these community clusters: CC_1_ has the lowest average out-degree in the mention network followed by CC_2_ and CC_3_. The clusters CC_3_ and CC_2_ are the most active; figure 9*a* shows that they consistently have the highest number of tweets per user. Cluster CC_3_ is the most tightly connected of the three, with a high transitivity coefficient in both the mention and follower networks. These community clusters are also consistently stratified by the sentiment of their tweets over time (figure 9*b*).

**Table 2.** Summary statistics for each community cluster. Note that the number of reciprocal links and transitivity are calculated for each community clusters network in isolation.

	CC_1_		CC_2_		CC_3_	
	mention	follower	mention	follower	mention	follower
users	1064		604		155	
links	32 076	85 302	22 333	45 799	8119	6409
reciprocal links	12 855	44 890	5527	22 171	2582	3163
avg. out-degree	30	80	37	76	52	41
transitivity	0.15	0.35	0.15	0.27	0.45	0.57

## 7. Support for the *yes* and *no* sides in the community clusters

Are the community clusters representative of *yes* or *no* supporters? To find out, we sample 358 (20%) users at random and manually classify them as either supporting *yes*, *no* or as *unaligned*. To classify each user, we examine their Twitter biography (self-description) and all their tweets in our dataset. If an account has no obvious leaning, such as an automated account (e.g. a *bot*), an institutional account or an impartial journalist, we classify it as *unaligned*. After classifying all the users in our sample, we examine the composition of each community cluster. Table 3 shows how the *yes*, *no* and *unaligned* users are distributed across the sample from each community cluster. See appendix B for a detailed outline of this procedure.

**Table 3.** Number of sampled *yes*, *no* and *unaligned* supporters in each community cluster.

		community cluster			
		CC_1_	CC_2_	CC_3_	total
alignment	*yes*	183	114	6	303
	*no*	1	2	23	26
	*unaligned*	21	5	3	29
	total	205	121	32	358

Users that support the *yes* side are predominantly found in community clusters CC_1_ and CC_2_ (89% of the users in CC_1_ and 96% of users in CC_2_ in the sample), while users that lean towards *no* are concentrated in CC_3_ (71% of the users in CC_3_ from the sample). Unaligned users are mostly found in CC_1_ and CC_3_. We categorize each community cluster according to the prevalence of *yes* and *no* leaning accounts; this achieves an accuracy of 89%, and a balanced accuracy [39] of 81% (see appendix B).

**Figure 10.**
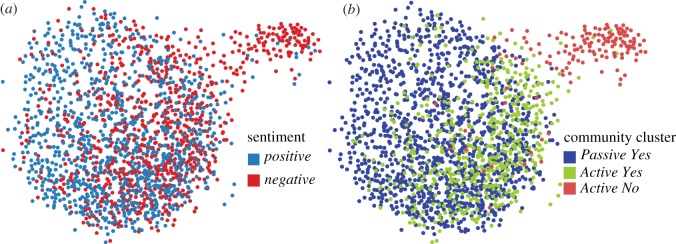
Reciprocal mention network in which the nodes are coloured by sentiment (*a*) and the final community-cluster partitions labelled by the side they support in the referendum (*b*). Edges removed for clarity.

As we observed in §6, the community clusters have varying levels of activity: members of CC_2_ and CC_3_ post twice as many mention tweets as CC_1_ over the observation period. Given these activity levels, and the distribution of support in table 3, we label community cluster CC_1_ as *Passive Yes*, CC_2_ as *Active Yes* and CC_3_ as *Active No*. Note that the total percentage of *no* supporters in the sample is 7%, while the referendum had a 40% *no* vote; this large difference is probably due to selection bias in the dataset. Note also the lack of a *Passive No* community cluster; its absence can be an artefact of the network construction in which we focused on reciprocal mentions (§4). Alternatively, it may be the case that less active *no* supporters did not engage or were absent from Twitter. Their absence will affect any interpretation of the interactions of *yes* and *no* supporters, where we are potentially missing a ‘silent’ cohort of *no* support. Figure 10 shows these classifications displayed on the layout of the mention network, alongside their sentiment.

### 7.1. Activity of community clusters

**Figure 11.**
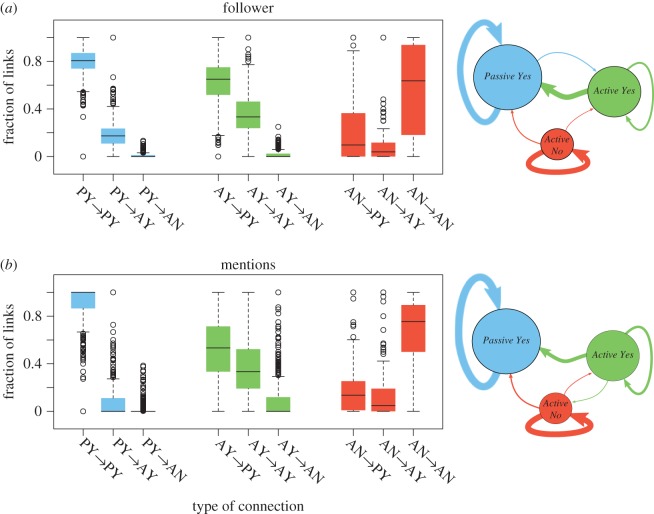
Fraction of connections between users in the three community clusters in the follower (*a*) and mention (*b*) networks.

We examine which community clusters interact more frequently through mentions and friend/follower links, the difference in the type of mention used (original, reply or retweet) and the sentiment of the interactions between community clusters. Figure 11*a* shows that users in the *Passive Yes* and *Active No* community clusters tend to follow mostly users within their own group (80% and 61% of user links, respectively), whereas users in the *Active Yes* cluster follow a disproportionately large number of users from *Passive Yes* (66%). Users in both *Yes* communities, on average, follow only a small number of users in *Active No* (0.7% and 2.6%, respectively). This pattern also appears in the mention network, where most connections are between members of the same community cluster (figure 11*b*). The strongest interaction between community clusters consists of connections between users in the *Yes* groups in both networks.

We also examine which type of mentions (original, replies or retweets) are used by the members of each group in their interactions. All community clusters retweet more often than they produce original messages or replies (table 4). Unsurprisingly, retweet connections occur most often between groups where there is already a high number of follower connections, as is the case with original mention tweets. Interestingly, reply tweets do not follow this trend; these messages tend to be sent to community clusters where there are few follower links to the source cluster. The users in the *Active Yes* and *Active No* community clusters produce the most reply tweets: 23% and 26% of their tweets are replies, respectively.

**Table 4.** Type of communication channel used between community clusters. Proportions are given for the total tweets originating from each group.

		mention tweets			proportion of the cluster’s tweets		
from	to	original	reply	retweet	original	reply	retweet
*Passive Yes*	*Passive Yes*	5302	1755	21 740	0.16	0.06	0.68
*Passive Yes*	*Active Yes*	206	306	2152	0.01	0.01	0.07
*Passive Yes*	*Active No*	139	168	308	0.00	0.00	0.01
*Active Yes*	*Passive Yes*	1200	1205	10 130	0.05	0.05	0.45
*Active Yes*	*Active Yes*	380	1935	4648	0.02	0.09	0.21
*Active Yes*	*Active No*	286	1948	601	0.01	0.09	0.03
*Active No*	*Passive Yes*	361	458	753	0.04	0.06	0.09
*Active No*	*Active Yes*	47	939	257	0.01	0.12	0.03
*Active No*	*Active No*	310	649	4345	0.04	0.08	0.54

The largest percentage of tweets between the *Active Yes* and *Active No* community clusters corresponds to replies (9% and 12%, respectively). This finding is surprising for two reasons. Firstly, there are very few follower connections between the two groups, which means that these messages bridged a gap between groups that do not typically interact. Secondly, these groups are ideologically opposed to each other. The *Passive Yes* community cluster, on the other hand, only sent 1.4% of its tweets in the form of replies to other community clusters. The two active *Yes* and *No* community clusters produced 73% of all replies, although they represent only 35% of all users.

We also calculate the fraction of original, replies and retweets that occurred in the presence of a follower link. Table 5 shows that of all the reply tweets between the active *Yes* and *No* communities, only 59% and 47% occurred when there was a follower link between the users. This is yet another indication that users in these two groups were more likely to engage with each other, even in the absence of strong structural ties. These results are consistent with the notion that although the marriage referendum was a heated topic on Twitter, the engagement between users with different views was limited to a small subset of highly active users. Note that because we only study tweets with at least one of the hashtags, it is possible that the actual number of replies was higher.

**Table 5.** Fraction of mention tweets that occurred between nodes that are connected in the follower network.

from	to	original	reply	retweet
*Passive Yes*	*Passive Yes*	0.99	0.96	0.97
*Passive Yes*	*Active Yes*	0.99	0.96	0.97
*Passive Yes*	*Active No*	0.68	0.49	0.53
*Active Yes*	*Passive Yes*	1.00	1.00	1.00
*Active Yes*	*Active Yes*	1.00	0.98	1.00
*Active Yes*	*Active No*	0.70	*0.59*	0.76
*Active No*	*Passive Yes*	0.96	0.81	0.75
*Active No*	*Active Yes*	0.83	*0.47*	0.60
*Active No*	*Active No*	0.94	0.76	0.97

**Figure 12.**
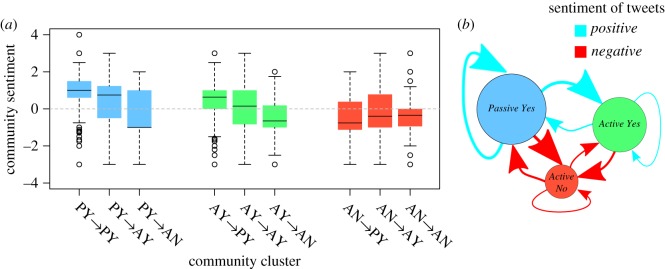
Box plots with the sentiment of the interactions between community clusters (*a*). On the network in (*b*), we see an illustration of these boxplots in the mention network. The size and colour of the arrow are proportional to the mean sentiment of the connections from each community cluster.

Given the differences in the type of mentions between the community clusters, we enquire whether the sentiment of the connections varies depending on the source and the target group. We unfold the average out-sentiment of each user (*S*_O_) to see the scores of tweets directed at each community cluster. Figure 12 shows that interactions with the Active No community cluster have, on average, more negative sentiment than other interactions. The interaction between the *Active Yes* and *Active No* clusters is overwhelmingly negative. Over 50% of users from both active community clusters use language with negative sentiment in their mention tweets sent between each other. The interactions of the *Passive Yes* cluster with itself, on the other hand, are overwhelmingly positive; over 50% have a positive sentiment score. The opposite is true for any interaction of the *Active Yes* with the *Active No* community cluster. This is the main feature that allows us to distinguish *Active Yes* from *Passive Yes*. The interactions between these two groups are almost all positive, and consist mostly of retweets. By contrast, the interactions between *Active Yes* and *Active No* are typically negative, and mostly consist of replies.

## 8. Conclusion

We have investigated the relationship between sentiment and social structure in the context of the Twitter discussion about the 2015 Irish Marriage referendum. We computed the sentiment scores of 204 626 tweets posted by 36 674 users, and constructed follower and mention networks among users in which the weight of the connections corresponds to the sentiment of the interactions. Although the sentiment score of individual tweets can be noisy, it can be aggregated successfully using networks to study the interactions between users in a mention and follower network. We performed extensive statistical tests to study the relationship between the sentiment of users’ tweets and their interactions, both in general (i.e. friend/follower) and topic-specifically (i.e. from tweets about the referendum). The correlation between the sentiment of mentions that a user sends and receives (the in- and out-sentiment) is positive and robust to randomization tests. Furthermore, users in the mentions network with positive and negative aggregate sentiment scores are more likely to be connected to users with similar sentiment than would occur by chance; positive users are also more likely to follow each other. The community structure of the networks shows that users with similar sentiment tend to be clustered together. By combining sentiment scores with the networks’ communities, we were able to find three distinct groups of users that we classified as either *yes* or *no* supporters based on the content of their tweets and sentiment, and as *active* or *passive* based on their activity. Interestingly, many of the mentions between the users in the *yes* and *no* groups occurred in the absence of friend/follower links, which indicates the existence of topical dialogue across ideological lines. These results show that sentiment and social structure are distinct yet related, and can be studied together to understand the disposition of users around topics of interest. This work can be extended in a number of directions, for example by combining sentiment analysis with topic modelling and additional user features (such as demographics, age, gender or income) to obtain a more accurate picture of user disposition. We anticipate that this work will also provide a basis for incorporating sentiment in opinion dynamics models, the analysis of retweet cascades, and to investigate the calibration of polling data using social structure.

## Figures and Tables

**Figure 13.**
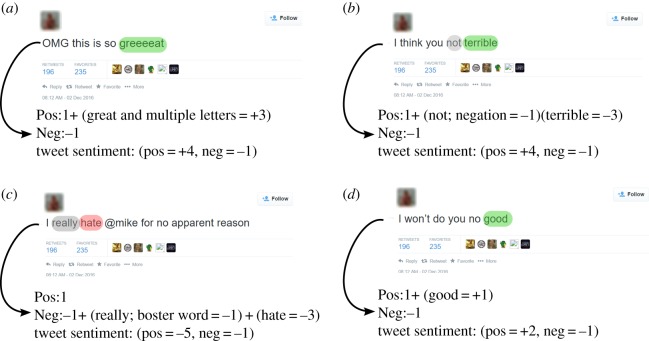
Examples of how SentiStrength assigns sentiment scores to various tweets. Highlighted words are detected as positive (green), negative (red), negations and boosters (grey). (*a*) Example of multiple letters. (*b*) Negation and booster words. (*d*) Failure to detect double negatives.

**Figure 14.**
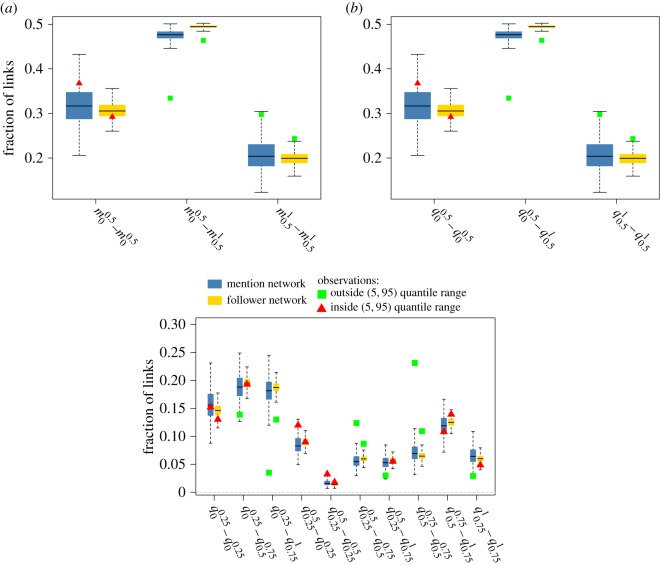
Results after 1000 randomization tests in the mention network and the follower network. (*a*) Division of users above and below the mean. (*b*) Division of users above and below the median. (*c*) Division of users into quantiles. Green squares and red triangles mark the observed fraction of links in the data. A green square indicates that the observed fraction falls outside the lower 5% and upper 95% quantiles of the randomized distribution. A red triangle indicates that observed fraction falls inside the lower 5% and upper 95% quantiles of the randomized distribution.

## Footnotes

1 https://gnip.com/realtime/powertrack/.

## Data accessibility

The IDs of the tweets used in this study are available in ReShare UK Data Service at URL: http://reshare.ukdataservice.ac.uk/852731/. With these tweets one can extract the text, compute sentiment, create retweet and mentions network. The follower network can be constructed by downloading the friend lists of the authors of the tweets.

## Appendix A. Sentiment extraction with SentiStrength

SentiStrength [33] contains a lexicon of 2310 sentiment-annotated words and word stems (i.e. roots of words). The system finds the sentiment of a string (more precisely a sentence or in this case a tweet) of text by matching each word against an internal lexicon. The positive and negative score of the string is obtained from its words, and is normalized to be between 1 and 5 for positive sentiment, and −1 and −5 for negative. SentiStrength also accounts for some nuances of the language by including an extensive rule set that includes negations, repeated letters (for emphasis) and booster words [33]. The rules for punctuation do not apply to our dataset as we removed punctuation as part of the pre-processing of the text. Figure 13 contains examples of how SentiStrength assigns positive and negative scores to short strings of text, and how in some cases it can miscalculate the sentiment of a tweet.

## Appendix B. Accuracy of the classification of users

The classification of community clusters as either *yes*, *no* or *unaligned* in §7 was performed manually by annotating a sample of 20% of users in each group. The classification of users was based on their profile description and their tweets with the tracked hashtags, and was blinded to the community cluster of the user. The profile descriptions are an indicator of which side users are likely to support, as they often contain hashtags, words or phrases in support of *yes* or *no* (e.g. #equalitymatters or #marriagematters can indicate support for *yes* or *no*). Tweets from the referendum day often contain references to having voted or supported yes or no (e.g. *I voted for equality #voteyes #marref*). In the absence of an overt reference to supporting either side, we classified the user after examining all their tweets in our data. We assigned an *unaligned* label if the user did not show a discernible leaning towards the *yes* or *no* side. Typically, users who were classified as unaligned either had posted few tweets or their tweets did not have an obvious leaning (e.g. *Interesting debate taking place now about #marref*).

After classifying the tweets from our random sample, we computed the proportion of *yes*, *no* or *unaligned* supporters in each community cluster. The proportion of *yes* supporters in community clusters CC_1_ and CC_2_ is 90% and 96%; as a result, we labelled these groups as *yes* community clusters. In CC_3_, about 71% of the sampled members support voting against same-sex marriage, so we labelled this community cluster as a *no* group. To find the accuracy of these labels, we construct a confusion matrix [39] (table 6), which provides a breakdown of true and false positives.

**Table 6.** Confusion matrix with the number of correct and incorrect classifications for yes and no voters.

		actual		
		yes	no	total
classification	*yes*	297 (true yes)	23 (true no)	320
	*no*	29 (false yes)	9 (false no)	38
	*total*	326	32	358

We can calculate the overall and balanced accuracy for *yes* and *no* supporters using table 6. The overall accuracy is the ratio of ‘true yes’ and ‘true no’ supporters (297 and 23, respectively) to the total number of users in the sample (358). The overall accuracy for the sample is 89%. However, overall accuracy is known to be biased towards more frequent classes. To correct for this bias, we obtain the *balanced accuracy* [39] by calculating the fraction of correctly classified *yes* or *no* supporters out of the total number of actual supporters, and averaging the two (297/326 and 23/32, respectively). The balanced accuracy is then (0.5(297/326 + 23/32) = 0.81).

In §7, we labelled the community clusters in terms of both the dominant user leaning and activity levels: CC_1_ as the *Passive Yes* community cluster; CC_2_, *Active Yes*; and CC_3_, *Active No*. In an ideal setting, we would report the balanced accuracy for the three types of users. In practice, however, it is a difficult and subjective exercise to discern *Passive Yes* from *Active Yes* users at an individual level, so distinction between passive and active is based on the average user’s activity (number of tweets) in each community cluster (figure 9*a*).

## Appendix C. Robustness of randomization

In §5, we showed that the sentiment of users’ in-neighbourhoods is positively correlated (in agreement with previous reports e.g. [17]) in both the mention and follower network; this finding allows us to use sentiment as a proxy for homophily. We arrived at this result by showing that users with similar sentiment, in particular positive users, were connected more often than we would have expected by random chance. These results are robust to distinct groupings of users by sentiment. Section 5 shows a coarse labelling of users according to their sentiment score (positive, negative or unknown). A finer labelling of users also produces similar results. We test three alternative ways of labelling users:

(i) Divide users into groups in which the out-sentiment is below ($m_{0}^{0.5}$) and above ($m_{0.5}^{1}$) the mean.
(ii) Divide users into groups in which the out-sentiment is below ($q_{0}^{0.5}$) and above ($q_{0.5}^{1}$) the median.
(iii) Divide users into groups by the out-sentiment quartiles ($q_{0}^{0.25}$, $q_{0.25}^{0.5}$, $q_{0.5}^{0.75}$, $q_{0.75}^{1}$).

We randomize the network with these labels in the same way as described in §5; figure 14 shows the results from the new randomization tests, which are consistent with our results in the main text. The similarity observed between figure 14*a* and *b* is due to the fact that the mean and median of the out-sentiment distribution are close. In both cases, users above the mean and median tend to be connected more than expected by chance. Figure 14*c* shows a similar story, where users in the top two quartiles are more likely to be connected with each other than what we would expect by chance.
